# Early Adolescent Predictors of Young Adults’ Distress and Adaptive Coping During the COVID-19 Pandemic: Findings From a Longitudinal Cohort Study

**DOI:** 10.1177/02724316231181660

**Published:** 2023-06-10

**Authors:** Annekatrin Steinhoff, Lydia Johnson-Ferguson, Laura Bechtiger, Aja Murray, Urs Hepp, Denis Ribeaud, Manuel Eisner, Lilly Shanahan

**Affiliations:** 1 Jacobs Center for Productive Youth Development, 27217 University of Zurich, Zurich, Switzerland; 2 University Hospital of Child and Adolescent Psychiatry and Psychotherapy, University of Bern, Bern, Switzerland; 3 Experimental and Clinical Pharmacopsychology, Department of Psychiatry, Psychotherapy, and Psychosomatics, Psychiatric University Hospital Zurich, 27217 University of Zurich, Zurich, Switzerland; 4 Department of Psychology, University of Edinburgh, Edinburgh, UK; 5 Meilen Institute Zurich, Zurich, Switzerland; 6 Institute of Criminology, 2152 University of Cambridge, Cambridge, UK; 7 Department of Psychology, 27217 University of Zurich, Zurich, Switzerland

**Keywords:** coping, stressful events, pandemic, longitudinal

## Abstract

We examined early adolescent predictors of later distress and adaptive coping in early adulthood, using data from a prospective longitudinal cohort study (*n* = 786). In early adolescence (age 13), we assessed indicators of mental health (internalizing symptoms), stressor exposure (cumulative stressful life events), and family socialization (supportive parent–child interactions). In early adulthood (age 22), during the first COVID-19-related Swiss national lockdown, we assessed cumulative pandemic-related stressors, distress (poor well-being, hopelessness, and perceived disruptions to life) and adaptive coping. Early adolescent internalizing symptoms predicted lower well-being, more hopelessness, and perceived lifestyle disruptions in early adulthood, during the pandemic. Cumulative stressful life events during early adolescence moderated the association between cumulative pandemic-related stressors and perceived lifestyle disruptions. Supportive parent–child interactions fostered subsequent engagement in adaptive coping, which, in turn, predicted less hopelessness and better well-being. Findings reveal that early adolescent development is linked with distress and adaptive coping in later periods.

## Introduction

Young people’s reactions to stressful events (e.g., emotional and cognitive-evaluative processes) and their adaptive coping responses play a vital role in mental health risk and resilience (i.e., better-than-expected outcomes given a particular level of exposure to adversity) (Horwitz et al., 2011; Vannucci et al., 2018). For example, experiencing declines in well-being and hope, and perceiving disruptions to one’s life due to a novel stressor (e.g., the Corona virus disease 2019 [COVID-19] pandemic), signal an increased risk of poor mental health (Folkman, 2010; Nearchou & Douglas, 2021; Shanahan et al., 2022). In turn, activating adaptive coping strategies can help maintain, restore, or improve mental health in the face of stressors (Compas et al., 2017).

Coping refers to the use of cognitive and behavioral strategies to manage the demands posed by stressors, including but not limited to regulating negative emotions (Compas et al., 2017; Folkman, 2012). Coping strategies can be self-sufficient (i.e., relying on oneself) or socially supported (i.e., involving others) (Litman, 2006). In addition, they can focus on emotions (e.g., self-distraction), meaning (e.g., cognitive reappraisal), and problem-solving (e.g., seeking contact with others when feeling lonely) (Hampel & Peterman, 2006; Schoenmakers et al., 2015). The effectiveness of coping strategies depends on the characteristics of the person and also of the stressful situation, including how much a situation can actually be changed and what exactly needs to be changed to decrease stress (Carroll, 2013; Tenenbaum et al., 2011).

In recent years, adolescents and young adults have faced a mental health crisis that has yet to be reversed (Gunnell et al., 2018; Office of the U.S. Surgeon General, 2021). An important step for remedying this crisis is to achieve a better understanding of the developmental antecedents that shape young people’s experiences of stress and their coping responses. However, developmental pathways towards distress and engagement in adaptive coping strategies are not well documented. Therefore, the key objective of this study is to examine early adolescent developmental precursors of distress and adaptive coping in early adulthood.

### Young adults’ distress and adaptive coping responses during the COVID-19 pandemic

The ways in which individuals experience stressful events and cope with them during the transition to adulthood can set the stage for their future mental and physical health and social functioning (Berg et al., 2017; Sloan et al., 2011). The COVID-19 pandemic and associated regulations (e.g., national lockdowns) had the potential to compound the stressors that are typically associated with the transition to adulthood (Shanahan et al., 2022; Steinhoff et al., 2021). In Spring 2020, the number of COVID-19 cases was increasing worldwide, including in Switzerland, our study site. Beginning in March, the Swiss government imposed “exceptional measures” (a so-called lockdown), prohibiting gatherings in larger groups; the closures of universities, shops, and borders; and work-at-home orders (Kohler et al., 2020). These restrictions likely conflicted with young adults’ strong desire to be engaged with social partners, particularly peers and romantic partners (Guarnieri et al., 2015). Furthermore, the work-at-home and remote study rules interfered with many young adults’ desire to make progress in their educational and occupational development (Shanahan et al., 2023).

When exposed to new stressors such as those associated with the COVID-19 pandemic, a decline in well-being and hope, and strong cognitions of life disruptions indicate a person’s initial distress. For example, hopelessness indicates a belief that the situation or stressor cannot be changed. These experiences and beliefs, in turn, can interfere with young adults’ mental health (Conner et al., 2001; Liu et al., 2015).

Adaptive coping strategies are strategies that buffer the potential negative effects of stress on well-being and functioning (Compas et al., 2017). The experience of successfully coping with stress can also strengthen a person’s sense of personal efficacy and investment into future goals (Shulman et al., 2009). Employing several different adaptive coping strategies (e.g., seeking social support, physical activity, self-distraction, and cognitive reappraisal) may offer the flexibility needed when facing life’s challenges, and, thus, increase a person’s likelihood of healthy adjustment (Cheng et al., 2014). This may be particularly relevant when a stressor affects multiple areas of life and limits personal freedoms.

For many people, the COVID-19 pandemic was an example of such a stressor. It threatened people’s personal health and that of close others. It also impacted people’s financial, educational, and professional situations and progress, and limited opportunities for social exchange and many leisure activities. These uncertainties and restrictions were often perceived as stressful; furthermore, they limited people’s opportunity to engage in their typical coping strategies of choice (Fegert et al., 2020; Shanahan et al., 2022). During such times, having a large repertoire of coping strategies, including social and self-sufficient ones (Litman, 2006), may be most adaptive. More specifically, maintaining social connections may be particularly adaptive, including by connecting in new ways (e.g., helping others in the neighborhood during the pandemic (Chen et al., 2021)). Furthermore, when news about the pandemic were almost omnipresent, cognitive strategies, such as positive reappraisal, and engagement in alternative activities for self-distraction may have been adaptive. Finally, in the context of home office regulations and widespread closures of leisure facilities, it may have been adaptive to keep up specific daily routines and raise one’s activity level where possible (e.g., by engaging in physical exercise) (Shanahan et al., 2022).

### Early Adolescent Precursors of Young Adult Distress and Adaptive Coping

During adolescence, young people have to master many new challenges, including in the biological (e.g., pubertal development), social (e.g., school transitions and expectations regarding educational success; new peer group dynamics), and psychological domains (e.g., emotional development). As they become increasingly independent and face stressors by themselves, early adolescents may begin to form typical patterns of experiencing and dealing with stress that they are likely to carry into the future (Gerber et al., 2013; Masselink et al., 2018; Ohannessian et al., 2010).

Early adolescents differ in how they experience and deal with stressors (Sheffler et al., 2019; Son et al., 2016; Yarcheski & Mahon, 2016). Relevant individual differences include mental health, levels of stressor exposure, and coping resources, such as supportive social environments that could model and teach adaptive coping strategies. These factors likely constitute developmental precursors of how young people will experience and deal with novel stressors in the future. In this study, we examine whether early adolescents’ internalizing symptoms, accumulation of stressful life events, and supportive parent–child interactions predict distress and adaptive coping strategies approximately one decade later, when faced with the COVID-19 pandemic in early adulthood.

*Internalizing symptoms*, such as anxiety and depressive symptoms, typically first occur in childhood or adolescence (Maughan et al., 2013) and signal an increased risk of various future mental health problems (Copeland et al., 2009; Rao & Chen, 2009). Internalizing symptoms can indicate previous exposure to and poor mastery of developmental challenges and stressful events, among many other factors. Indeed, internalizing symptoms are often a manifestation of one’s difficulty in regulating negative emotions in daily life and the inability to effectively engage in adaptive coping in response to stress (Son et al., 2016). In turn, internalizing problems are associated with future engagement in maladaptive strategies of dealing with stress, such as rumination (Calvete et al., 2015).

*Exposure to stressful life events* during early adolescence can play different roles in future distress and adaptive coping. On the one hand, cumulative stressful events can have a sensitizing effect (Rutter, 2012) by triggering strong stress responses that influence one’s physiological stress systems in lasting ways and thus set the stage for future internalizing mental health problems (Low et al., 2012; Steinhoff et al., 2020). Consistent with this evidence, research has shown that exposure to stressful life events, especially their accumulation, casts a “long shadow” and increases people’s later vulnerability to novel stressors (Appleyard et al., 2005; Liu et al., 2014). For example, severe stress in childhood (e.g., maltreatment) is associated with poorer emotion regulation years later (Gruhn & Compas, 2020; Tottenham et al., 2010). Furthermore, the risk of psychiatric disorders following stress exposure in adulthood is increased among those with a history of childhood adversity (McLaughlin et al., 2010).

On the other hand, exposure to stressful life events in early adolescence could prepare young people for future stress (i.e., an inoculation effect) (Rutter, 2012). For example, according to one study, adolescents who had mastered stress associated with adverse experiences in childhood in a healthy way had a decreased risk of mental health problems when facing novel stressors (Oldehinkel et al., 2014). Potential mechanisms underlying such associations include that the first experience of successfully mastering stress could *decrease* adolescents’ sensitivity to future stressful events (e.g., by decreasing their hopelessness or perception of disruptions due to novel stressors). Furthermore, exposure to stressful events provides the opportunity to practice and increase one’s repertoire of adaptive coping strategies. For example, early adolescents may be able to actively seek and cultivate extra-familial support when facing a stressful event; such social networks during stressful times can be re-activated (or replicated in other settings) during subsequent periods of stress (Spitz et al., 2020).

A *supportive home environment* is a known resilience factor in adolescent mental health development (Rueger & Malecki, 2011). Research has shown that parents who respond to their children’s negative emotions in supportive ways foster their children’s emotion regulation abilities (Perry et al., 2020; Spinrad et al., 2020), which could be among the most important developmental foundations for coping with novel stressors (Compas et al., 2017). Indeed, parents can explicitly encourage and scaffold or implicitly model and socialize the use of coping strategies (Tu et al., 2020; Zimmer-Gembeck & Locke, 2007). In homes where parents frequently engage in supportive conversations with their children and offer emotional and practical support when needed, adolescents may learn that social support is generally available and useful. As adolescents develop, they may continue to employ these skills and attitudes and also transfer them to peers and partners (Skinner & Zimmer-Gembeck, 2007). Supportive parent–child interactions that involve joint activities can also foster children’s general (physical) activity level (Gustafson & Rhodes, 2006) and their perspective-taking skills (Hall et al., 2021). In turn, specific adaptive coping strategies may be developed, such as engaging in physical or other activities, including for self-distraction, or for evaluating stressors from different perspectives and, potentially, reframing it in a positive light (i.e., positive reappraisal).

### The Current Study

**Figure 1.**
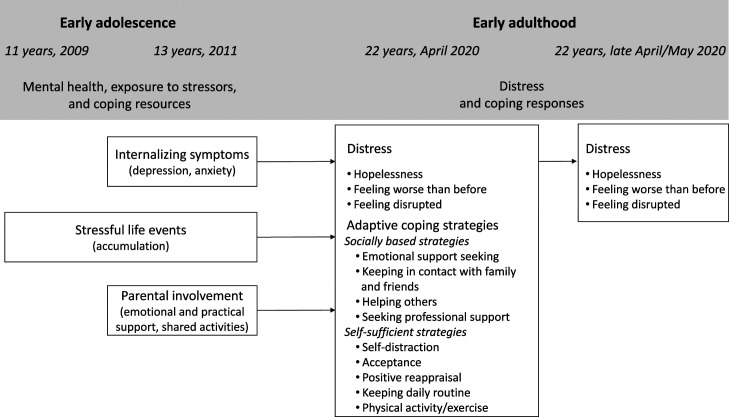
Conceptual framework: early adolescent predictors of distress and adaptive coping in early adulthood. *Note.* Interaction effects between the early adolescent factors and cumulative pandemic-related stressors and indirect effects of early adolescent predictors on early adulthood distress via adaptive coping were also explored.

We leveraged data from a community-based prospective-longitudinal study to investigate how internalizing symptoms, cumulative stressful life events, and supportive parent–child interactions in early adolescence predicted young adults’ distress and adaptive coping during the first months of the COVID-19 pandemic. Figure 1 illustrates our study design.

Our young adult outcome variables were distress surrounding the pandemic and lockdown in early adulthood (i.e., feeling worse, hopelessness, and perceiving disruptions to one’s life), and the frequency of the use of adaptive coping strategies. We discuss our expectations regarding associations between each of our predictors and these outcome variables in turn.

Our general hypotheses were: 1) Early adolescent internalizing symptoms are associated with increased levels of feeling worse, hopelessness, and perceived lifestyle disruptions during the pandemic. 2) Early adolescents’ exposure to cumulative stressful life events either increase (i.e., sensitization hypothesis) or mitigate (i.e., inoculation hypothesis) the distress. 3) Supportive parent–child interactions in early adolescence are associated with decreased levels of all indicators of later distress. Regarding coping, we assumed that 4) early adolescent internalizing symptoms signal an increased risk of not engaging in adaptive coping strategies in early adulthood, whereas 5) the exposure to stressful life events might encourage adolescents to learn and engage in adaptive coping strategies. Finally, we hypothesized that 6) a higher frequency of supportive parent–child interactions in early adolescence is associated with increased engagement in adaptive coping in early adulthood. We explored associations of the early adolescent predictors and specific adaptive coping strategies in a follow-up analysis. Furthermore, to explore the actual adaptiveness of the coping strategies considered, we examined whether the early adolescent predictors are indirectly associated with subsequent reductions of distress via coping as assessed here.

We adjusted all models for the adolescents’ household socio-economic status (SES), parental migration background, and sex, because prior research has linked these factors with the emotional processing of and coping with stressful events (Buckley et al., 2019; Eschenbeck et al., 2018; Taylor & Stanton, 2007). We also adjusted for the individual exposure to cumulative pandemic-related stressors (e.g., job loss and disease), which are known predictors of mental health during the pandemic (Robillard et al., 2021; Shanahan et al., 2022; Steinhoff et al., 2021) and, thus, also of distress and adaptive coping. Finally, considering that not everybody was affected by the pandemic in the same way, we explored whether the impact of the early adolescent factors on distress during the pandemic varied as a function of the individual’s exposure to pandemic-related stressors (i.e., interaction effects between the early adolescent factors and cumulative pandemic-related stressors).

## Methods

### Sample and procedures

We used data from the Swiss longitudinal community-representative Zurich Project on the Social Development from Childhood to Adulthood (z-proso) (Ribeaud et al., 2022). The initial target sample included 1,675 children who entered first grade at one of 56 public primary schools in Zurich, Switzerland’s largest city, in 2004. The schools were selected using stratified random sampling procedures, with slight oversampling of disadvantaged school districts.

The participants were assessed eight times between ages 7 (*n* = 1,360) and 20 (in 2018; *n* = 1,180). Those who participated in 2018 were subsequently invited to participate in four additional waves of data collection between April and September of 2020 (i.e., during the first months of the COVID-19 pandemic in Switzerland). At that time, the participants were approximately 22 years old. The main variables used in the present paper come from the age 13 assessment (in 2011; *n* = 1,365), representing early adolescence, and the first during-pandemic assessment, in April 2020, when the participants were ∼22 years old. In addition, we used distress indicators assessed in late April/early May 2020 (i.e., the second during-pandemic assessment) in our follow-up analyses. We included all respondents who participated in at least the April 2020 assessment (*n* = 786). To draw inferences from the analytic sample to the original study sample (i.e., the approximately representative sample of first-graders in Zurich in 2004), we applied survey weights in all parts of the analyses (i.e., descriptive statistics and multivariate modeling). The survey weights have been described in detail elsewhere (Nivette et al., 2021).

At the age of 13, the participants completed paper-and-pencil questionnaires in a classroom setting, which lasted about 90 minutes (details on study procedures can be found in Ribeaud et al., 2022). In 2020, during the pandemic, the participants completed an online survey lasting approximately 15–20 minutes and were given 7 days to complete this survey. At age 13, the participants received a ∼$30 cash incentive; in 2020, they were entered into a lottery to win one of 50 prizes of ∼$100. The participants provided written informed consent for their study participation at age 13; parents could opt their children out of the study. In 2020, at age 22, the participants signed an online informed consent. Ethical approval was obtained from the Ethics Committee of the Faculty of Arts and Social Sciences of the University of Zurich. All procedures involved in this work comply with the ethical standards of the relevant national and institutional committees on human experimentation and with the Helsinki Declaration of 1975, as revised in 2008.

### Variables

#### Early Adulthood Outcomes

*Distress* was assessed twice, first between April 8 and 15 and then again between April 30 and May 5, 2020. We asked the respondents “How much worse or better do you feel since the beginning of the Corona pandemic?”, “How hopeful are you about the future?”, and “How much has the Corona pandemic disrupted your lifestyle, thinking about your daily routines, work, education, and family?” The participants answered each item using a 10-point scale (i.e., 1 = much worse/not at all hopeful/high level of lifestyle disruptions to 10 = much better/very hopeful/no lifestyle disruptions). For the analyses, we recoded these scales, so that higher values indicate feeling worse, more hopeless, and more disrupted.

*Adaptive coping strategies* were assessed at the first during-pandemic assessment. We asked the participants how often they had engaged in specific activities when having experienced something stressful during the previous two weeks (see Online Supplement for complete wording of the assessment). The overall framing did not explicitly refer to the current pandemic as the relevant stressor, thus leaving open whether the participants had used the strategies when feeling stressed because of the pandemic itself, other reasons, or both. However, the timeframe (i.e., previous two weeks) referred to the pandemic timeframe only.

Some items explicitly referred to the pandemic, the wording of others was more general. Items adapted from Carver (1997) were partly reformulated to explicitly capture coping strategies relevant during the COVID-19 pandemic and included acceptance of the pandemic as something real and trying to find something good in the pandemic (i.e., positive reappraisal). Other items from Carver were not adapted to specifically capture pandemic-related coping, including engaging in activities for self-distraction, seeking emotional support, and maintaining contact with close others. In addition to the Carver items, we created items to assess strategies that might be particularly relevant during a pandemic and lockdown. Specifically, we asked how often the participants engaged in keeping a daily routine, physical exercise, helping others in the neighborhood, and seeking professional mental health support (e.g., via the telephone). The participants rated the frequency of their engagement in each of these strategies on a four-point scale (1 = never, 4 = very often). Inter-item correlations were low/non-significant to moderate (see Table S1, online supplement) and internal consistency among all items was relatively low (Cronbach’s α = .54). This may, in part, be due to the different contextualization of the items (i.e., some explicitly referring to stress due to the pandemic and others referring to any kind of stress) and suggests that the participants engaged in several different sub-sets of coping strategies.

To assess overall coping flexibility (Cheng et al., 2014), we created a sum score to indicate the overall frequency of any adaptive coping. Summing the different coping strategies that an individual has used is similar to the approach taken in cumulative risk research (Appleyard et al., 2005) and reflects the variety of coping strategies used. Being based on the full frequency scales instead of dichotomized items (as is usually the case in cumulative risk research), however, our score also reflects the average frequency of using each strategy (e.g., a score of 21 could indicate that a participant has engaged in a total of seven strategies, each at a moderate frequency, or that they have engaged in only four strategies frequently). Thus, the score also accounts for coping preferences (i.e., using one strategy at a high frequency counts as much as using different strategies at a low frequency each).

We also examined the predictors of specific coping strategies in follow-up analyses. In these analyses, we used a) the original single items and b) two summary scores indicating flexibility in the domains of social and self-sufficient coping, respectively (Litman, 2006). To indicate the use of socially based strategies, we combined (i.e., summed) items referring to seeking emotional support from others, maintaining contact with close others, helping others in the neighborhood, and seeking professional support. To indicate the use of self-sufficient strategies, we combined self-distraction, acceptance of the pandemic, positive reappraisal, keeping a daily routine, and physical exercise.

#### Early Adolescent Predictors

*Internalizing symptoms* during the previous month were assessed using eight items from the Social Behavior Questionnaire (Tremblay et al., 1991). The items address depressive and anxiety symptoms (e.g., I felt sad without reason; I felt anxious). Participants reported how often these symptoms had occurred, using a five-point scale (1 = never, 5 = very often). We created a mean scale (α = .82), with higher values indicating a higher level of internalizing symptoms.

##### Cumulative stressful life events

The 13-year-olds were presented with a list of 25 life events and asked to indicate which ones had occurred since the last assessment (i.e., since the 11-year-old assessment two years ago). We selected 21 events that were likely perceived as stressful (e.g., death of a close person or of a pet, parental separation, hospitalization). We created a sum score indicating how many events had occurred between the ages of 11 and 13. In other words, we captured life events during much of the early adolescent period.

##### Supportive parent–child interactions

We used six items from the parental involvement subscale of the Alabama Parenting Questionnaire (Shelton et al., 1996). The items refer to parental emotional and practical support (e.g., my parents comfort me, I can ask my parents for help if I have a problem; my parents help me with my homework) and a family climate characterized by frequent joint activities (e.g., talking, playing, or doing other things together). The participants reported how often these things generally occurred at home using a four-point scale (1 = never, 4 = very often). We combined the items (α = .72) by creating a mean score, with higher values indicating more supportive parent–child interactions.

#### Control and Context Variables

We measured *SES* using the International Socio-Economic Index (Ganzeboom et al., 1992). This score ranges from 16 (e.g., unskilled workers) to 90 (e.g., judges). We used the maximum score achieved at any assessment during adolescence (ages 11–15) and then reversed the scale, with higher values indicating lower adolescent SES. We used binary assessments of *parental migration background* (1 = both parents born abroad, 0 = at least one parent born in Switzerland) and *sex* recorded at birth (female = 1, male = 0).

*Cumulative stressors related to the COVID-19 pandemic and lockdown.* At the April 2020 assessment, we asked the participants whether they had experienced several events since the beginning of the pandemic. We selected 15 events that would likely be stressful and created a sum score indicating how many different events had occurred (Steinhoff et al., 2021). This score includes health-related (e.g., exposure to COVID-19) and economic/educational events (e.g., job loss and financial difficulties) that happened to the participants themselves (e.g., hospitalization due to COVID-19 symptoms) or close others (e.g., death of a close person due to COVID-19 and parental unemployment) because of the pandemic or lockdown.

### Analytic strategy

We specified linear regression models to assess the associations of the early adolescent predictors (i.e., internalizing symptoms, cumulative stressful life events, and supportive parent–child interactions) and the young adult indicators of distress and adaptive coping during the first COVID-19 lockdown (outcomes assessed at the first during-pandemic assessment). For hypothesis testing, we first assessed the adjusted associations between the early adolescent predictors and the outcomes in separate models (i.e., one model specified for each predictor and outcome, controlling for socio-economic background, migration background, and sex). We then included all three early adolescent predictors in the same model (i.e., full model) to assess their unique associations with the young adult outcomes. In the full models, we adjusted for socio-demographics and cumulative pandemic-related stressors, to account for individual differences in actual stressor exposure and associated impairments of well-being (Robillard et al., 2021; Shanahan et al., 2022; Steinhoff et al., 2021). We explored two-way interactions between the early adolescent predictors and cumulative pandemic-related stressors on distress. We centered the variables involved in the interaction terms and entered one interaction term at-a-time into the full models. The models were specified in MPlus V8 using a maximum likelihood estimator robust to non-normality (MLR) (Muthén & Muthén, 2017).

To examine indirect effects of early adolescent predictors on distress at the second during-pandemic assessment via coping at the first during-pandemic assessment (i.e., our follow-up analyses), we specified path models. We used the MODEL INDIRECT command in MPlus and computed bias-corrected bootstrapped standard errors (1000 draws) (Hayes, 2009; MacKinnon et al., 2004). The effects of coping on the distress outcomes were controlled for socio-demographics, cumulative pandemic-related stressors, and the respective distress indicator at the first during-pandemic assessment (see online supplement, Tables S3a, S4a, and S5a for more details). MLR was not available in mediation models with bootstrapping and therefore maximum likelihood (ML) estimation was used.

For each of the age 22 variables assessed in April 2020 (first during-pandemic assessment), 1–2% of participants had missing data; for each of the age 13 variables, about 5% of participants had missing data. For the variables assessed in late April/early May 2020 (second during-pandemic assessment), 18% of participants had missing data, mainly due to sample attrition. Although Little’s missing completely at random (MCAR) test was not significant (*p* = 0.059), we used state-of-the-art methods to reduce potential bias due to selective missing data. Specifically, we conducted multiple imputation of missing values (Enders, 2013; Schafer & Graham, 2002). Missing values on predictor and outcome variables were imputed (Bayesian estimation as implemented in MPlus) from an unrestricted model that included all variables involved in the main analyses (i.e., all variables included here, except the late April/May assessments of distress) and the survey weights. Missing data on the single items for adaptive coping strategies were imputed, and the sum score to indicate overall adaptive coping frequency was then computed based on the complete data. We imputed 20 data sets and report averaged estimates and pooled standard errors across these data sets (Rubin, 1987). In the mediation models involving the late April/May assessments of distress, full information maximum likelihood was used, since these models were not available with multiple imputation.

## Results

Table 1 shows descriptive statistics for and correlations among the main study variables. Although feeling worse, hopelessness, and perceiving lifestyle disruptions in early adulthood were positively correlated with each other, the small to moderate magnitude of these correlations indicates that the three items assess different aspects of the participants’ distress during the COVID-19 pandemic.

**Table 1.** Descriptive Statistics and Correlations of Main Study Variables.

	Mean (SD)	1	2	3	4	5	6	7	8	9	10
1. Low socio-economic status (adolescence)	43.05 (20.02)	—									
2. Parental migration background	- ^a^	0.48***	—								
3. Female sex	- ^b^	0.02	0.01	—							
4. Internalizing symptoms (13 years)	2.20 (0.72)	−0.07	−0.09*	0.32***	—						
5. Cumulative stressful life events (13 years)	2.66 (1.82)	0.07	0.01	0.04	0.23***	—					
6. Supportive parent–child interactions (13 years)	3.09 (0.56)	−0.28***	−0.23***	0.06	−0.15***	−0.09*	—				
7. Cumulative pandemic-related stressors^c^ (22 years)	0.92 (1.13)	0.06	0.03	0.01	0.10*	0.08	−0.13**	—			
8. Feeling worse, April 2020^c^ (22 years)	5.80 (1.48)	0.01	0.02	−0.03	0.08*	0.06	−0.03	0.04	—		
9. Hopelessness, April 2020^c^ (22 years)	4.12 (2.03)	−0.05	−0.02	0.13***	0.14***	−0.02	−0.03	0.00	0.22***	—	
10. Lifestyle disruptions, April 2020^c^ (22 years)	6.28 (2.44)	−0.08*	−0.02	0.12**	0.12**	0.02	0.07	0.14***	0.29***	0.14***	—
11. Frequency of any adaptive coping, April 2020^c^ (22 years)	21.61 (3.61)^d^	−0.19***	−0.11**	0.19***	0.02	0.01	0.20***	0.02	−0.15***	−0.14***	−0.03

*Note.* MLR estimation was used. Descriptive statistics for distress indicators assessed in late April/May 2020 and their correlations with the main study variables can be found in the online supplement. * *p* < 0.05, ** *p* < 0.01, and *** *p* < 0.001.

^a^Participants whose parents were both born abroad: 51%.

^b^Female participants: 48%.

^c^First during-pandemic assessment.

^d^Statistics for a mean score instead of a sum score: M = 2.40 (SD = 0.40).

### Early Adolescent Precursors of Distress in Early Adulthood

The separate and full regression models (Table 2) consistently showed that internalizing symptoms in early adolescence were a main predictor of all three indicators of the young adults’ distress during the first months of the COVID-19 pandemic and lockdown. Specifically, higher levels of internalizing symptoms at age 13 were associated with feeling worse than before the pandemic, more hopeless, and perceiving more lifestyle disruptions. There were no main effects of early adolescent exposure to cumulative stressful life events or supportive parent–child interactions on these outcomes. Female participants reported more hopelessness and perceived lifestyle disruptions than male participants, and cumulative pandemic-related stressors were also associated with the perception of lifestyle disruptions.

**Table 2.** Early Adolescent Predictors of Distress and Adaptive Coping in Early Adulthood: Standardized Coefficients From Separate and Full Regression Models (in Which All Predictors Were Entered Simultaneously).

Predictors	Feeling worse, April 2020				Hopelessness, April 2020			
	Separate models^a^		Full model^b^		Separate models^a^		Full model^b^	
	*β*	*p*	*β*	*p*	*β*	*p*	*β*	*p*
Socio-demographics								
Low socio-economic status	0.00	0.995	0.00	0.978	−0.05	0.276	−0.05	0.293
Parental migration background	0.02	0.657	0.03	0.548	0.00	0.957	0.00	0.920
Female sex	−0.03	0.363	−0.06	0.106	**0.13**	<0.001	**0.10**	0.007
Early adolescence								
Internalizing symptoms	**0.10**	0.014	**0.09**	0.042	**0.10**	0.010	**0.11**	0.011
Cumulative stressful life events	0.06	0.155	0.04	0.405	−0.02	0.611	−0.05	0.256
Supportive parent–child interactions	−0.03	0.487	0.00	0.963	−0.06	0.188	−0.04	0.387
Early adulthood								
Cumulative pandemic-related stressors	0.04	0.284	0.03	0.422	0.01	0.898	−0.01	0.882
	Lifestyle disruptions, April 2020				Frequency of any adaptive coping, April 2020			
	Separate models^a^		Full model^b^		Separate models^a^		Full model^b^	
Predictors	*β*	*p*	*β*	*p*	*β*	*p*	*β*	*p*
Socio-demographics								
Low socio-economic status	**−0.10**	0.026	−0.09	0.056	**−0.18**	<0.001	**−0.15**	<0.001
Parental migration background	0.03	0.459	0.05	0.259	−0.03	0.461	−0.02	0.698
Female sex	**0.12**	0.001	**0.09**	0.023	**0.20**	<0.001	**0.20**	<0.001
Early adolescence								
Internalizing symptoms	**0.09**	0.020	**0.09**	0.027	−0.06	0.115	−0.04	0.311
Cumulative stressful life events	0.03	0.507	0.00	0.976	0.01	0.783	0.03	0.511
Supportive parent–child interactions	0.04	0.320	0.08	0.062	**0.14**	<0.001	**0.14**	0.001
Early adulthood								
Cumulative pandemic-related stressors	**0.15**	<0.001	**0.15**	<0.001	0.03	0.491	0.05	0.293

^a^For each predictor, a separate model was specified. All models adjusted for socio-demographics.

^b^All predictors were included in the same model, controlling for socio-demographics.

*Note.* Bold print indicates significant effects (*p* < 0.05).

Our exploration of interaction effects between the early adolescent predictors and later cumulative pandemic-related stressors revealed that exposure to cumulative stressful life events in early adolescence moderated the effect of pandemic-related stressors on perceived lifestyle disruptions (*β* = −0.11, *p* = 0.003). Figure S1 (online supplement) provides an illustration of this moderation, showing that the size of the effect of cumulative pandemic-related stressors on perceived lifestyle disruptions decreased with an increasing number of stressful life events experienced during early adolescence. In other words, exposure to a higher number of stressful live events in early adolescence shielded young adults, to some extent, from feeling disrupted when encountering novel stressors. This supports the inoculation hypothesis. The figure also shows that, among young adults who had experienced 1.5 or more stressful life events above the average in early adolescence, cumulative pandemic-related stressors were not associated with an increased risk of feeling disrupted (i.e., the 95% confidence intervals of the effect sizes include zero). Other interaction effects between early adolescent factors and cumulative pandemic-related stressors were not significant (*p* > 0.05).

### Early Adolescent Precursors of Adaptive Coping in Early Adulthood

The separate and full models consistently showed that higher levels of supportive parent–child interactions in early adolescence were associated with more frequent use of any adaptive coping strategies in early adulthood (Table 2), whereas effects of early adolescent internalizing symptoms and cumulative stressful life events were not significant. Furthermore, lower SES was associated with a lower frequency of adaptive coping, and female sex was associated with a higher frequency of adaptive coping.

In follow-up exploratory analyses, we examined whether the associations between the early adolescent predictors and early adult adaptive coping differ when the individual adaptive coping strategies and the summary scales indicating social and self-sufficient strategies are examined as the outcomes, instead of the overall sum score of adaptive coping frequency. The results from the full models including the single coping items and all three early adolescent predictors are shown in Table 3. Supportive parent–child interactions in early adolescence were associated with increased engagement in several adaptive coping strategies that involved social interaction (i.e., seeking emotional support, maintaining contact with family and friends, helping others in the neighborhood) in young adulthood. In addition, early adolescent supportive parent–child interactions were associated with more engagement in cognitive reappraisal (i.e., finding something good in the current situation). Early adolescent exposure to cumulative stressful life events and internalizing symptoms were not associated with any individual adaptive coping strategy in early adulthood. Finally, the models that included the summary scores of self-sufficient and social coping strategies as outcomes confirmed that supportive parent–child interactions in early adolescence predicted a higher frequency of using self-sufficient strategies (*β* = 0.09, *p* = 0.030) and, especially, socially based strategies (*β* = 0.15, *p* < 0.001). Other early adolescent predictors were not significantly associated with the summary scores.

**Table 3.** Early Adolescent Predictors of Individual Adaptive Coping Strategies in Early Adulthood: Standardized Coefficients From Full Regression Models.

	Seeking emotional support		Contact with family/friends		Helping others^a^	
Predictors	*β*	*p*	*β*	*p*	*β*	*p*
Socio-demographics						
Low socio-economic status	−0.01	0.845	**−0.09**	0.045	0.01	0.807
Parental migration background	−0.04	0.288	−0.02	0.740	−0.02	0.652
Female sex	**0.23**	<0.001	**0.09**	0.018	0.06	0.109
Early adolescence						
Internalizing symptoms	0.00	0.991	−0.08	0.069	−0.05	0.237
Cumulative stressful life events	0.00	0.973	0.06	0.147	0.01	0.886
Supportive parent–child interactions	**0.11**	0.002	**0.11**	0.012	**0.11**	0.006
Early adulthood						
Cumulative pandemic-related stressors	**0.11**	0.007	−0.01	0.728	0.06	0.154
	Seeking professional support^a^		Self-distraction		Acceptance	
Predictors	*β*	*p*	*β*	*p*	*β*	*p*
Socio-demographics						
Low socio-economic status	0.01	0.759	−0.03	0.583	**−0.13**	0.003
Parental migration background	0.04	0.374	0.02	0.588	0.01	0.782
Female sex	**−0.08**	0.042	**0.17**	<0.001	0.02	0.606
Early adolescence						
Internalizing symptoms	0.07	0.117	0.04	0.388	0.01	0.890
Cumulative stressful life events	0.01	0.841	−0.01	0.877	0.00	0.970
Supportive parent–child interactions	−0.03	0.570	0.02	0.684	0.03	0.555
Early adulthood						
Cumulative pandemic-related stressors	**0.14**	0.024	**0.11**	0.011	0.00	0.989
	Cognitive reappraisal		Keeping a daily routine		Physical activity/exercise	
Predictors	*β*	*p*	*β*	*p*	*β*	*p*
Socio-demographics						
Low socio-economic status	**−0.10**	0.017	−0.04	0.325	**−0.18**	<0.001
Parental migration background	0.08	0.050	−0.04	0.421	**−0.09**	0.028
Female sex	0.07	0.089	**0.13**	0.001	0.06	0.099
Early adolescence						
Internalizing symptoms	−0.01	0.901	−0.05	0.261	−0.06	0.132
Cumulative stressful life events	0.08	0.064	−0.03	0.478	−0.01	0.904
Supportive parent–child interactions	**0.10**	0.019	0.05	0.217	0.06	0.104
Early adulthood						
Cumulative pandemic-related stressors	0.03	0.595	**−0.10**	0.006	−0.06	0.124

*Note.* Bold print indicates significant effects (*p* < 0.05).

^a^For consistency with previous analyses (Shanahan et al., 2022), a sensitivity analysis with these outcomes being dichotomized was carried out. These showed similar associations between the early adolescent factors and the respective coping strategy (i.e., significant effect of supportive parent–child interactions on helping others; no significant associations with seeking professional support).

### Follow-Up Analyses of Indirect Effects of Early Adolescent Predictors On Distress Via Coping

The path models revealed that adaptive coping during the first months of the pandemic was associated with decreased levels of feeling worse and hopelessness a few weeks later (adjusted coefficients for feeling worse: *β* = −0.10, *p* = 0.029; hopelessness: *β* = −0.11, *p* = 0.011; *n* = 694). The adjusted association between adaptive coping and perceived lifestyle disruptions was not significant (*β* = −0.03, *p* = 0.426). Although supportive parent–child interactions in early adolescence were not directly associated with the distress outcomes at the second during-pandemic assessment, the path models indicated significant indirect negative effects of supportive parent–child interactions in early adolescence on feeling worse (unstandardized *b* = −0.05, 95% CI from −0.11 to −0.01) and hopelessness (unstandardized *b* = −0.06, 95%CI from −0.12 to −0.02) through adaptive coping. Other results from these models can be found in the online supplement (Tables S3a, S3b, S4a, S4b, S5a, S5b).

## Discussion

A better understanding of the developmental precursors of distress and adaptive coping is important for reversing the current mental health crisis among young people (Gunnell et al., 2018; Office of the U.S. Surgeon General, 2021). Our study reveals that early adolescent mental health, stressor exposure, and coping resources in the family each play a role in predicting early adults’ distress and adaptive coping. Early adolescent internalizing symptoms predicted young adults’ distress, while early adolescent exposure to stressful life events buffered the impact of novel cumulative during-pandemic stressors on select distress outcomes. Supportive parent–child interactions predicted young people’s later engagement in adaptive coping strategies, which were, in turn, associated with decreased levels of specific forms of distress.

### The Role of Early Adolescent Internalizing Symptoms

The associations of early adolescent internalizing symptoms and distress almost one decade later during the COVID-19 pandemic likely reflect the continuity of mental health over time (Copeland et al., 2009; Rao & Chen, 2009), and also that early adolescents with a greater tendency to internalize problems are vulnerable when exposed to novel stressors in subsequent years. To illuminate these developmental processes further, future research should investigate whether late-adolescent internalizing problems mediate the association between early adolescent symptoms and distress in early adulthood.

But why were early adolescents’ internalizing symptoms not associated with less adaptive coping during young adulthood? One possible reason is that internalizing problems are associated with more frequent use of maladaptive, unhealthy strategies to deal with stress (e.g., rumination and self-harm) (Krause et al., 2018; Nock et al., 2006) in addition to, and not necessarily instead of, trying adaptive strategies. Future research is needed to investigate the association of early adolescent mental health and subsequent *patterns of maladaptive and adaptive strategies* in the face of stress. Another explanation could be that adolescents with internalizing symptoms may have had the opportunity to learn about and practice adaptive coping strategies. This could have included the use of mental health services by some (Wu et al., 2001). If that was the case, positive and negative associations of internalizing symptoms with subsequent adaptive coping strategies for different subgroups in this sample (e.g., those who received support and were able to develop healthy strategies for emotion regulation vs. those who kept suffering emotion dysregulation) could have counterbalanced each other to produce a null effect.

### The Role of Stressful Life Events in Early Adolescence

Our data provided some support for the stress inoculation hypothesis (Rutter, 2012). Early adults who had been exposed to an unusually high number of stressful life events in their early adolescence had no increased risk of experiencing lifestyle disruptions when facing novel, pandemic-related stressors. One putative mechanism involved in these associations could be that adolescents who have gone through many stressful life events in the past develop a different cognitive framing of how to interpret changes in life compared to those who have not been exposed to a considerable number of stressful life events. In other words, the number of additional life events needed to be perceived as *disruptions* to life could be relatively high among those who have been exposed to many stressful events during previous developmental periods.

Notably, there were no main effects of early adolescent exposure to stressful life events and no interaction effects with cumulative pandemic-related stressors with regard to any of the other indicators of distress (i.e., hopelessness or feeling worse). This could be due to our assessments focusing on the mere exposure to stressful life events in early adolescence, without assessing how participants appraised and mastered them. In fact, those who did not successfully cope with or overcome previous stressful life events may be at the highest risk of negative distress when facing novel stressors, whereas only those who mastered the previous events successfully and in a healthy manner may have benefited from the previous stressful experiences (Oldehinkel et al., 2014). Therefore, to investigate the inoculation vs. sensitization hypotheses further, additional information on the mastery of these prior events may be indispensable.

### The Role of Supportive Parent–Child Interactions in Early Adolescence

Our data indicate that supportive parent–child interactions in early adolescence are not directly linked with early adult distress but support the acquisition of adaptive coping strategies that can be actively used into adulthood. These findings provide new insights into the long-term developmental processes towards coping and distress. Specifically, our findings indicate that supportive parent–child interactions in early adolescence are indirectly associated with attenuated distress (see also Ford et al., 2023) in the form of hopelessness and feeling worse when encountering a novel stressor, one decade later, by fostering adaptive coping. Previous research mostly focused on the effectiveness of coping strategies in alleviating distress (Buhle et al., 2013; Wang et al., 2021), and the therapeutic approaches suitable for supporting the necessary adaptive coping skills (Regehr et al., 2013; Yeo et al., 2020), but less on whether adaptive coping processes can be fostered earlier in life, within family settings.

Our follow-up analyses showed that family socialization in particular played a role when it came to adaptive coping strategies involving social interactions, including seeking support *from* others and providing it *to* others. Early adults likely seek support from various sources besides their parents, including friends and partners (Skinner & Zimmer-Gembeck, 2007). In fact, at the age 22 assessment, about 30% of our sample had moved out of the parental home, mostly to apartments shared with peers or partners (Steinhoff et al., 2021; the relatively high proportion of young adults still living in the parental home is consistent with the Swiss context of high housing costs.) During the lockdown, the people living in one’s household were likely among the main sources of social support. Therefore, our findings suggest that parents who facilitate frequent exchange about daily problems and offer their children emotional and practical support in early adolescence may have a lasting impact on the children’s capacity to seek social support not only from parents but also from other relationship partners.

Family socialization was also a significant predictor of self-sufficient coping strategies, although this association was less robust than the association between family socialization and social coping strategies (i.e., family socialization was not associated with all self-sufficient strategies considered). In the association between supportive parent–child interactions and cognitive reappraisal, a potential underlying mechanism could be the young adults’ ability to take different perspectives on a stressful situation (Hall et al., 2021) and thus their ability to find something good in the pandemic despite the negative things they might also see in it. Another mechanism could be the long-term associations between parent–child interactions in adolescence and the quality of relationships with parents, peers, and partners in early adulthood (Overbeek et al., 2007). While social gatherings involving larger groups were prohibited during the lockdown, rules about working from home, remote studying, and the closure of various out-of-home leisure facilities (e.g., sports facilities) likely led to more time spent with select close others, especially those in the same household. For those who regularly enjoyed these social interactions, it might have been easier to reappraise (i.e., reframe) the pandemic and lockdown positively. In fact, the opportunity to spend more time with close others was among the most frequently mentioned positive aspects of pandemic life among young adults in this sample (Shanahan et al., 2023).

### Limitations and Future Directions

The list of adaptive coping strategies considered here was limited and additional strategies (e.g., making plans; humor; or emotion expression, suppression, and modulation [Compas et al., 2017]) might have revealed additional associations with the early adolescent predictors. In fact, our measurement of coping was limited by assessing specific strategies with single items and relatively low overall internal consistency. Still, we were able to measure a variety of adaptive coping strategies, including those that may work irrespective of the particular stressor (e.g., self-distraction and acceptance) and others that were likely specifically relevant during the COVID-19 pandemic and lockdown (e.g., helping others in the neighborhood and keeping a daily routine). Indeed, our results showed that coping, as assessed here, preceded decreases in hopelessness and increases in well-being during the first months of the pandemic.

Testing several hypotheses simultaneously and specifying several models that include different measures of adaptive coping (i.e., a sum score versus separate models including the single items) comes with the limitations of multiple testing. However, we used the rather conservative approach of two-tailed *p*-values despite having mostly directional hypotheses; all effects we identified were in the hypothesized directions; and the main findings were linked to a *p*-value that would indicate statistical significance even if corrections for alpha-error-inflation were applied. Specifically, when we use a Bonferroni-corrected *p*-value of 0.05/3 = 0.016 for hypothesis testing in the realm of distress, which reflects that three indicators of distress were used as outcomes, the significant association between early adolescent internalizing symptoms and hopelessness remains. Furthermore, when we apply a *p*-value of 0.05/10 = 0.005, which accounts for 10 tests involving different coping measures as outcomes, the association between early adolescent supportive parent–child interactions and the overall adaptive coping score in the main model remains significant.

Given that supportive parent–child interactions were not associated with later distress on the bivariate level in our sample, despite the significant indirect paths via adaptive coping, our findings illustrate the complexity of links between early adolescent development, adaptive coping, and mental health in early adulthood. To fully understand these links, future research should ideally consider mediating and moderating processes occurring during mid- and late-adolescence (e.g., mental health trajectories, additional stressors encountered in mid- and late-adolescence, or extra-familial coping resources, including supportive social interactions in schools [García-Carrión et al., 2019]). This was beyond the scope of the current paper, however, which focuses on identifying potential early adolescent starting points of such trajectories.

The effect sizes in our models were relatively small but especially given that almost a decade had passed between the assessments, they were remarkable (Götz et al., 2022). Still, additional research is needed to examine the role of other early adolescent factors in the development of distress and adaptive coping across a lifetime. For example, explicit measures of strategies used for every-day emotion regulation in early adolescence could be promising target concepts. Alexithymia (i.e., difficulty in recognizing, understanding, and verbalizing one’s emotions) could be another one, assuming that recognizing one’s emotional state could be an important precursor of engaging in adaptive coping strategies (Khan & Jaffee, 2022). Future research should also investigate the mechanisms underlying the correlations of SES and sex with adaptive coping frequency and several specific adaptive coping strategies, which were evident in several of our models.

## Conclusions

The transitions to adolescence and adulthood are marked by significant development in the biological, cognitive, emotional, and social domains. During these periods, the ways in which young people experience and cope with stress can change fundamentally (Aldwin, 2012; Romeo, 2010; Skinner & Zimmer-Gembeck, 2007). Nevertheless, our study shows that early adolescent development and socialization are linked with distress and adaptive coping responses in early adulthood. Monitoring early adolescent mental health could help identify young people at risk of ongoing negative distress until adulthood. Intervention mechanisms targeting supportive parent–child interactions, for example, by fostering parental support and shared activities in families, could improve adolescents’ capacities for adaptive coping across their lifetimes.

## Supplemental Material

Supplemental Material - Early Adolescent Predictors of Young Adults’ Distress and Adaptive Coping During the COVID-19 Pandemic: Findings From a Longitudinal Cohort Study Click here for additional data file. Supplemental Material for Early Adolescent Predictors of Young Adults’ Distress and Adaptive Coping During the COVID-19 Pandemic: Findings From a Longitudinal Cohort Study by Annekatrin Steinhoff, Lydia Johnson-Ferguson, Laura Bechtiger, Aja Murray, Urs Hepp, Denis Ribeaud, Manuel Eisner, and Lilly Shanahan in the Journal of Early Adolescence.
