# Supplementary material for: Early Adolescent Predictors of Young Adults’ Distress and Adaptive Coping During the COVID-19 Pandemic: Findings From a Longitudinal Cohort Study
Source: J Early Adolesc. 2023 Jun 10;44(9):1250–80. doi: 10.1177/02724316231181660 (PMC10261967; doi:10.1177/02724316231181660)
Supplement: Supplemental Material - Early Adolescent Predictors of Young Adults’ Distress and Adaptive Coping During the COVID-19 Pandemic: Findings From a Longitudinal Cohort Study [file sj-pdf-1-jea-10.1177_02724316231181660.pdf]

Online Supplement

Early adolescent predictors of young adults' distress and adaptive coping during the COVID-19 pandemic: Findings from a longitudinal cohort study

Annekatrin Steinhoff, Lydia Johnson-Ferguson, Laura Bechtiger, Aja Murray, Urs Hepp, Denis Ribeaud, Manuel Eisner, & Lilly Shanahan

Coping assessment

Items adapted from (Carver, 1997) and additional items created by z-proso study team; see also Shanahan et al. (2022)

Item stem: There are lots of ways to try to deal with stress. The next few questions ask you to indicate how you dealt with stress in the past 2 weeks. There are no "right" or "wrong" answers. In the past two weeks, how often have you done the following things when you experienced something stressful?

- I have been seeking emotional support from others (e.g. from family, friends).
- I have been distracting myself with other things to take my mind off things (e.g. watching TV or films, reading, daydreaming, sleeping, playing with my pet, online shopping, cooking, cleaning, etc.)
- I have been accepting the reality that the corona crisis is real.
- I have been trying to find something good in the corona crisis (e.g. that that due to the crisis my relationship with a person that is important to me has become stronger).
- I have exercised or engaged in physical activity.
- I have been involved in helping neighbors during the corona crisis (e.g. went grocery shopping or picked up medicine for elderly/at-risk persons).
- I have been keeping in contact with my family, other close persons, and friends.
- I have been trying to keep up a daily routine.
- I have been seeking help at a telephone helpline, help chat, or from a counseling service or psychotherapist.

Note. The study questionnaire was administered in German. In instances where items were originally in English (but had been translated into German), we show the original English wording. For all other items, we show ad hoc translations of the original German items into English.

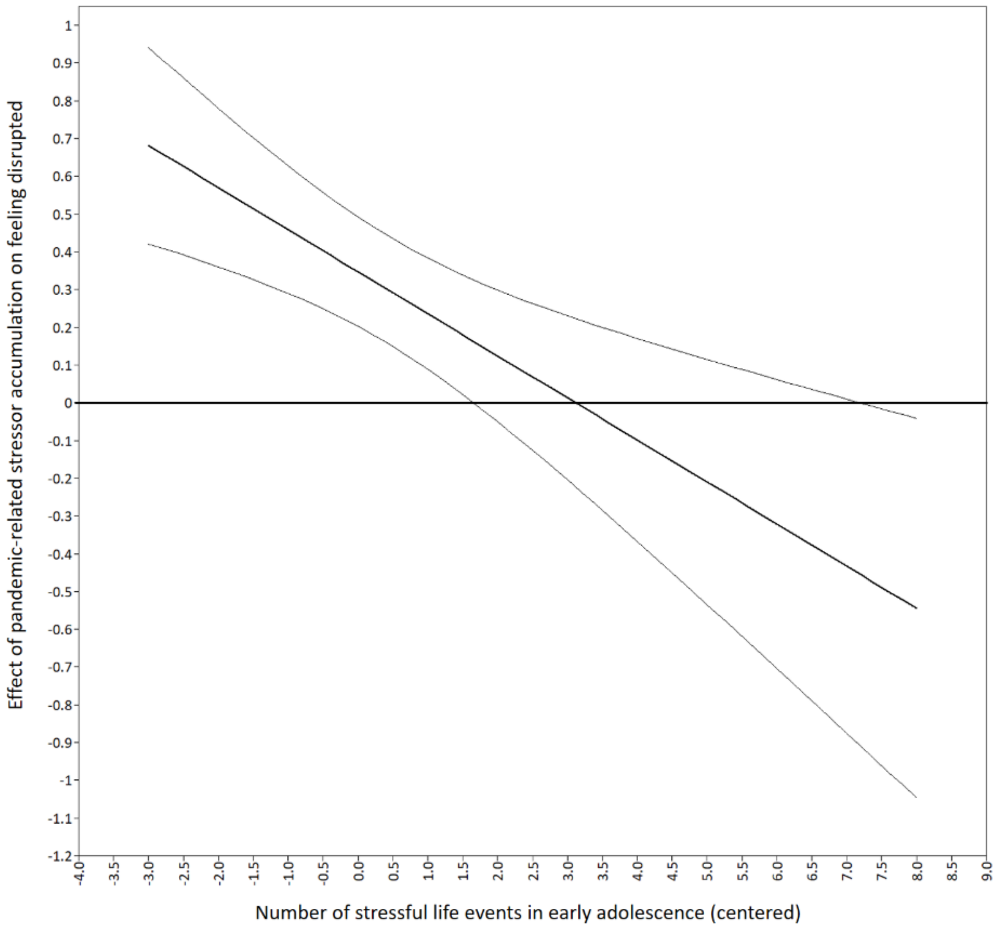

Figure S1. The number of stressful events in early adolescence moderates the effect of pandemic-related stressor accumulation on early adults' perceived lifestyle disruptions: unstandardized coefficients and their 95% confidence intervals.

Table S1. Descriptive statistics and correlations: adaptive coping strategies.

	Mean (SD)	1.	2.	3.	4.	5.	6.	7.	8.
1. Seeking emotional support	2.07 (0.89)	-							
2. Self-distraction	2.90 (0.88)	0.28***	-						
3. Acceptance	3.14 (0.83)	0.03	0.15***	-					
4. Cognitive reappraisal	2.66 (1.04)	0.16***	0.18***	0.26***	-				
5. Physical activity/exercise	2.54 (1.05)	0.04	0.03	0.11**	0.19***	-			
6. Helping others	1.46 (0.76)	0.11**	0.01	0.07*	0.13***	0.14***	-		
7. Contact with family/friends	2.98 (0.83)	0.17***	0.10*	0.09*	0.25***	0.15***	0.13***	-	
8. Keeping a daily routine	2.76 (0.90)	0.10**	-0.05	0.15***	0.23***	0.27***	0.08*	0.31***	-
9. Seeking professional support	1.12 (0.47)	0.14**	0.02	-0.08	-0.08	-0.09*	0.03	-0.08	-0.04

Note: * $p < 0.05$, ** $p < 0.01$, *** $p < 0.001$.

Table S2. Descriptive statistics and correlations: distress at the late April/May 2020 assessment.

	Distress in late April/May 2020		
	Feeling worse	Hopelessness	Lifestyle disruptions
Mean (SD)	5.48 (1.67)	4.19 (1.99)	5.79 (2.51)
Correlations with...			
1. Low socio-economic status (adolescence)	-0.08	-0.02	-0.11**
2. Parental migration background	-0.05	-0.00	-0.02
3. Female sex	-0.02	0.15***	0.14***
4. Internalizing symptoms (13 years)	0.05	0.14***	0.19***
5. Cumulative stressful life events (13 years)	-0.08	0.01	0.06
6. Supportive parent-child interactions (13 years)	0.05	0.01	0.06
7. Cumulative pandemic-related stressors (22 years)	0.05	-0.00	0.16***
8. Feeling worse, April 2020 (22 years)	0.38***	0.21***	0.23***
9. Hopelessness, April 2020 (22 years)	0.15***	0.54***	0.18***
10. Lifestyle disruptions, April 2020 (22 years)	0.12**	0.19***	0.51***
11. Frequency of any adaptive coping, April 2020 (22 years)	-0.10*	-0.15***	0.02

Table S3a. Path model specifications and results: standardized coefficients from model with feeling worse in late April/May 2020 as the final outcome.

	Standardized estimate	***p***
**Regressions**		
Feeling worse, late April/May 2020 on…		
Low socio-economic background, adolescence	-0.05	0.295
Parental migration background	-0.04	0.372
Sex (female)	0.00	0.981
Internalizing symptoms, early adolescence	0.07	0.166
Cumulative stressful life events, early adolescence	-0.08	0.069
Supportive parent-child interactions, early adolescence	0.07	0.144
Cumulative pandemic-related stressors, April 2020	0.05	0.320
Feeling worse, April 2020	0.36	< 0.001
Adaptive coping, April 2020	-0.10	0.029
Adaptive coping, April 2020 on…		
Low socio-economic background, adolescence	-0.14	0.001
Parental migration background	-0.04	0.362
Sex (female)	0.19	< 0.001
Internalizing symptoms, early adolescence	-0.04	0.334
Cumulative stressful life events, early adolescence	0.04	0.275
Supportive parent-child interactions, early adolescence	0.15	< 0.001
Cumulative pandemic-related stressors, April 2020	0.06	0.164

Table S3b. Indirect paths from early adolescent predictors to feeling worse in late April/May 2020 through adaptive coping in April 2020.

**Early adolescent predictor**	**Unstandardized coefficient (95% CI)**
Internalizing symptoms	0.01 (from -0.00 to 0.05)
Cumulative stressful life events	-0.00 (from -0.02 to 0.00)
Supportive parent-child interactions	-0.05 (from -0.11 to -0.01)

Table S4. Path model specifications and results: standardized coefficients from model with hopelessness in late April/May 2020 as the final outcome.

	Standardized estimate	***p***
**Regressions**		
Hopelessness, late April/May 2020 on…		
Low socio-economic background, adolescence	-0.04	0.251
Parental migration background	0.02	0.576
Sex (female)	0.08	0.052
Internalizing symptoms, early adolescence	0.07	0.076
Cumulative stressful life events, early adolescence	0.02	0.564
Supportive parent-child interactions, early adolescence	0.05	0.205
Cumulative pandemic-related stressors, April 2020	0.00	0.925
Hopelessness, April 2020	0.49	< 0.001
Adaptive coping, April 2020	-0.11	0.011
Adaptive coping, April 2020 on…		
Low socio-economic background, adolescence	-0.14	0.001
Parental migration background	-0.04	0.363
Sex (female)	0.19	< 0.001
Internalizing symptoms, early adolescence	-0.04	0.339
Cumulative stressful life events, early adolescence	0.04	0.275
Supportive parent-child interactions, early adolescence	0.15	< 0.001
Cumulative pandemic-related stressors, April 2020	0.06	0.167

Table S4b. Indirect paths from early adolescent predictors to hopelessness in late April/May 2020 through adaptive coping in April 2020.

**Early adolescent predictor**	**Unstandardized coefficient (95% CI)**
Internalizing symptoms	0.01 (from -0.01 to 0.05)
Cumulative stressful life events	-0.01 (from -0.02 to 0.00)
Supportive parent-child interactions	-0.06 (from -0.12 to -0.02)

Table S5. Path model specifications and results: standardized coefficients from model with perceived lifestyle disruptions in late April/May 2020 as the final outcome.

	Standardized estimate	***p***
**Regressions**		
Lifestyle disruptions, late April/May 2020 on…		
Low socio-economic background, adolescence	-0.09	0.022
Parental migration background	0.03	0.444
Sex (female)	0.07	0.080
Internalizing symptoms, early adolescence	0.11	0.007
Cumulative stressful life events, early adolescence	0.04	0.263
Supportive parent-child interactions, early adolescence	0.05	0.212
Cumulative pandemic-related stressors, April 2020	0.08	0.045
Lifestyle disruptions, April 2020	0.46	< 0.001
Adaptive coping, April 2020	-0.03	0.426
Adaptive coping, April 2020 on…		
Low socio-economic background, adolescence	-0.14	< 0.001
Parental migration background	-0.04	0.373
Sex (female)	0.19	< 0.001
Internalizing symptoms, early adolescence	-0.04	0.325
Cumulative stressful life events, early adolescence	0.04	0.274
Supportive parent-child interactions, early adolescence	0.15	< 0.001
Cumulative pandemic-related stressors, April 2020	0.06	0.172

Table S5b. Indirect paths from early adolescent predictors to perceived lifestyle disruptions in late April/May 2020 through adaptive coping in April 2020.

**Early adolescent predictor**	**Unstandardized coefficient (95% CI)**
Internalizing symptoms	0.00 (from -0.01 to 0.04)
Cumulative stressful life events	-0.00 (from -0.01 to 0.00)
Supportive parent-child interactions	-0.02 (from -0.09 to 0.02)

References

Carver, C. S. (1997). You want to measure coping but your protocol's too long: consider the brief COPE. *International Journal of Behavioral Medicine*, *4*(1), 92-100. doi:10.1207/s15327558ijbm0401_6

Shanahan, L., Steinhoff, A., Bechtiger, L., Murray, A. L., Nivette, A., Hepp, U., . . . Eisner, M. (2022). Emotional distress in young adults during the COVID-19 pandemic: evidence of risk and resilience from a longitudinal cohort study. *Psychological Medicine*, *52*(5), 824-833. doi:10.1017/s003329172000241x
